# Exposição ao metilmercúrio e neurodesenvolvimento em crianças indígenas: um modelo teórico

**DOI:** 10.1590/0102-311XPT228623

**Published:** 2025-04-11

**Authors:** Adriana Duringer Jacques, Breno Augusto Bormann de Souza, Carlos Augusto Ferreira de Andrade, Juliana dos Santos Vaz, Paulo Cesar Basta

**Affiliations:** 1 Escola Nacional de Saúde Pública Sergio Arouca, Fundação Oswaldo Cruz, Rio de Janeiro, Brasil.; 2 Departamento de Saúde Coletiva, Universidade Federal do Rio Grande do Norte, Natal, Brasil.; 3 Faculdade de Nutrição, Universidade Federal de Pelotas, Pelotas, Brasil.

**Keywords:** Mercúrio, Transtornos do Neurodesenvolvimento, Causalidade, Povos Indígenas, Mercury, Neurodevelopmental Disorders, Causality, Indigenous Peoples, Mercurio, Trastornos del Neurodesarollo, Causalidad, Pueblos Indígenas

## Abstract

O neurodesenvolvimento é um processo que se estende desde a concepção até a idade adulta, abrangendo o crescimento e a maturação do sistema nervoso. As crianças indígenas que vivem na Região Amazônica enfrentam diversas condições que podem impactar adversamente seu neurodesenvolvimento, incluindo assistência pré-natal, condições de nascimento, desnutrição, presença de doenças infecciosas e desafios socioeconômicos. A exposição a substâncias tóxicas durante o período pré-natal é mais um elemento dos determinantes que influenciam o neurodesenvolvimento. No caso do metilmercúrio, a exposição é associada ao consumo materno de peixes contaminados. Após o nascimento, a criança continua a ser exposta pela amamentação ou alimentação. Este ensaio teve como objetivo elaborar um modelo teórico para elucidar os determinantes do neurodesenvolvimento nos primeiros 1.000 dias de vida, considerando a exposição pré-natal ao metilmercúrio em crianças indígenas. Para a elaboração do modelo teórico, cumpriu-se as sete etapas propostas pelo *Checklist para Relato Teórico em Estudos Epidemiológicos*. Apresentamos reflexões teórico-metodológicas sobre dimensões e variáveis explicativas que destacam como a exposição a condições adversas desde a vida intrauterina é agravada pela presença do metilmercúrio, podendo resultar em prejuízo à saúde. Este modelo teórico é inédito ao considerar a complexa rede causal envolvida e contribui para pesquisas e ações que implementam a saúde materno-infantil.

## Abstract (en)

Neurodevelopment spans from conception to adulthood, encompassing the growth and maturation of the nervous system. Indigenous children living in the Amazon face several circumstances that can adversely impact their neurodevelopment, including prenatal care, birth conditions, malnutrition, infectious diseases, and socioeconomic challenges. Prenatal exposure to toxic substances configures another determinant influencing neurodevelopment. Exposure to methylmercury is associated with the maternal consumption of contaminated fish. After birth, children continue to undergo exposure by breastfeeding or feeding. This trial aimed to develop a theoretical model to find the determinants of neurodevelopment in the first 1,000 days of life considering prenatal exposure to methylmercury in Indigenous children. The seven steps of the *Checklist for Theoretical Report in Epidemiological Studies* were followed to elaborate this theoretical model. We offer theoretical-methodological reflections on the dimensions and explanatory variables that highlight how exposure to adverse conditions since intrauterine life worsens in the presence of methylmercury, which can damage human health. This theoretical model is unprecedented in considering the complex causal network involved and contributes to research and actions on maternal and child health.

## Resumen

El neurodesarrollo es un proceso que se extiende desde la concepción hasta la edad adulta abarcando el crecimiento y la maduración del sistema nervioso. Los niños indígenas que viven en la Región Amazónica están enfrentando diversas situaciones que pueden afectar negativamente su desarrollo neurológico, incluida la atención prenatal, las condiciones de nacimiento, la desnutrición, la presencia de enfermedades infecciosas y los desafíos socioeconómicos. La exposición a sustancias tóxicas durante el período prenatal es otro elemento de los determinantes que influyen en el neurodesarrollo. En el caso del metilmercurio, la exposición está asociada al consumo materno de pescado contaminado. Después del nacimiento, el niño sigue expuesto con la lactancia materna o la alimentación. Este ensayo tuvo como objetivo desarrollar un modelo teórico para revelar los determinantes del neurodesarrollo en los primeros 1.000 días de vida, teniendo en cuenta la exposición prenatal al metilmercurio en niños indígenas. Para elaborar un modelo teórico se cumplieron los siete pasos propuestos por la *Lista de Verificación para el Informe Teórico en Estudios Epidemiológicos*. Se presentan reflexiones teórico-metodológicas sobre dimensiones y variables explicativas que destacan cómo la exposición a condiciones adversas desde la vida intrauterina se ve agravada por la presencia del metilmercurio, lo que puede ocasionar daños a la salud. Este modelo teórico no tiene precedentes por considerar la compleja red causal involucrada y contribuye a la investigación y las acciones relacionadas con la salud maternoinfantil.

## Introdução

Nas últimas décadas, o avanço na assistência à saúde da mulher e da criança, aliado à melhora da qualidade de vida da população, proporcionou redução expressiva nas taxas de mortalidade infantil ^1^. Em consequência, ações de assistência e cuidado à saúde continuam sendo incentivadas, entre elas, a vigilância do neurodesenvolvimento tem apresentado uma progressiva relevância ^2^ ^,^ ^3^ ^,^ ^4^. Por meio de intervenções adequadas, criam-se condições para que a criança alcance o seu máximo potencial de crescimento e desenvolvimento ^2^ ^,^ ^3^ ^,^ ^4^.

O neurodesenvolvimento refere-se ao processo de desenvolvimento do sistema nervoso, desde a concepção até a idade adulta, ocorrendo principalmente durante a gestação e nos primeiros anos de vida. Envolve a formação e organização das estruturas do cérebro, bem como o desenvolvimento das funções motoras e sensoriais, das funções cognitivas, emocionais e comportamentais ^3^. Os primeiros mil dias de vida constituem uma janela de oportunidade para o desenvolvimento humano. Durante esse período, o cérebro e o corpo da criança crescem e se desenvolvem rapidamente, moldados por fatores genéticos, ambientais e sociais ^5^. As alterações do neurodesenvolvimento podem ser causadas em diferentes períodos: durante o período pré-natal, durante o parto, ou após o nascimento ^3^.

Fatores ambientais como a exposição a metais pesados igualmente podem ter efeitos nocivos para o desenvolvimento infantil. A atividade de extração de minerais do subsolo ou solo para a comercialização, notadamente o garimpo de ouro, libera resíduos de mercúrio nos rios e contamina os peixes, que constituem uma das principais fontes alimentares para os indígenas ^6^. O metilmercúrio (MeHg), compreendido como a forma orgânica resultante da metilação do mercúrio inorgânico no leito dos rios, é uma substância tóxica para o feto e para a criança nos primeiros meses de vida ^6^. No Brasil, por consequência do garimpo ilegal, o MeHg pode ser encontrado no ambiente aquático da Amazônia, a região mais pesquisada nesse contexto ^7^ ^,^ ^8^ ^,^ ^9^. A instalação ilegal de garimpeiros em terras indígenas para a extração de ouro trouxe consequências para a saúde desses povos, bem como prejuízos para a manutenção de sua cultura e tradição. Além de afetar a saúde dos povos originários, o mercúrio é um poluente ambiental, pois contamina o solo, a água e o ar, causando danos à flora, à fauna e ao clima ^8^ ^,^ ^9^. 

A principal via de exposição humana ao MeHg se dá por meio do consumo de peixes contaminados ^10^. O MeHg presente nos rios contamina algas, plantas, peixes e outros animais aquáticos. Dessa forma, esses peixes acabam sendo consumidos por populações ribeirinhas e indígenas da região, provocando múltiplos danos à saúde ^10^. 

A exposição pré-natal à substância provoca alterações bioquímicas e estruturais no sistema nervoso central do feto, que resultam no atraso do neurodesenvolvimento ^11^. Estudos recentes reportam associação entre a exposição intrauterina ao MeHg e alterações do neurodesenvolvimento em indígenas na Amazônia ^7,8,9,12^. Um dos estudos demonstrou que 25% das crianças avaliadas em área de exposição ao MeHg apresentavam atraso de neurodesenvolvimento ^12^. O estudo também revelou a alta prevalência de exposição crônica ao MeHg em mulheres em idade fértil ^12^. Ademais, o metilmercúrio pode estar presente no leite materno de nutrizes contaminadas, e no peixe consumido pela criança a partir da introdução alimentar complementar após os primeiros meses de vida ^13^.

Historicamente, as populações indígenas no Brasil enfrentam diversas ameaças à saúde, muitas vezes decorrentes de pressões exercidas pela grilagem de terras, pelo agronegócio, pela instalação de hidrelétricas, pela indústria extrativista de madeira, e pelo garimpo. A invasão dos territórios indígenas compromete seriamente a segurança alimentar e a saúde desses povos ^9^. O *Inquérito Nacional de Saúde e Nutrição dos Povos Indígenas* realizado no Brasil, entre 2008 e 2009, avaliou a situação de saúde de crianças e mulheres indígenas em idade reprodutiva ^14^. O estudo revelou que na época mais da metade (51%) das crianças indígenas sofriam de anemia e 27,5% apresentavam déficit de crescimento ^14^. Esse cenário é atribuído a múltiplas causas, incluindo condições inadequadas de vida, alimentação insuficiente em nutrientes essenciais, falta de acesso a cuidados pré-natais de qualidade, além de altas prevalências de doenças infecciosas, e da exposição a substâncias tóxicas, como o MeHg ^15^. Portanto, a exposição crônica ao metilmercúrio constitui fator de risco adicional para a saúde de gestantes e crianças indígenas ^16^.

Apesar do conhecimento sobre os efeitos do MeHg no neurodesenvolvimento, não foram encontrados estudos que explorem modelos teóricos e variáveis relacionadas ao impacto em crianças indígenas no Brasil e no mundo ^17^ ^,^ ^18^. Os modelos teóricos ajudam a organizar conhecimentos e entender relações complexas entre variáveis ^(^ ^18^, além de facilitar a investigação dos mecanismos envolvidos e a interpretação dos resultados ^(^ ^17^. Eles também permitem testar hipóteses com dados clínicos, socioeconômicos, biomarcadores e testes neuropsicológicos, essenciais para avaliar o efeito do MeHg no desenvolvimento infantil ^(^ ^17^ ^,^ ^18^.

A interação entre essas variáveis pode acrescentar elementos relevantes no contexto estudado ^19^. Para exemplificar, a exposição ao MeHg pode ser acompanhada por complicações, como prematuridade e baixo peso ao nascer. Dois estudos demonstraram que o aumento de duas vezes no nível de mercúrio no sangue do cordão umbilical mostrou-se associado a uma redução significativa do peso ao nascer e do perímetro cefálico ao nascer ^20^ ^,^ ^21^. Assim, além do mecanismo direto de lesão neurológica decorrente do MeHg, a prematuridade e o baixo peso ao nascimento também provocam danos neurológicos. Por outro lado, o estudo revelou que a associação entre o MeHg e o neurodesenvolvimento foi modificada pela presença de ácidos graxos poli-insaturados das séries ômega-3 e 6 presentes nos peixes ^21^. Ou seja, nesse caso, o alimento contaminado contém elementos de proteção, como ácidos graxos poli-insaturados, ferro, iodo, selênio, colinas e vitaminas ^21^.

Assim, para desenvolver estratégias de prevenção e intervenção eficazes a fim de garantir o pleno neurodesenvolvimento infantil de crianças indígenas, é necessário compreender os fatores que o influenciam. A determinação desses fatores envolve a elucidação de componentes biológicos, culturais, ambientais e socioeconômicos. Dessa maneira, levando-se em conta o risco de exposição ao MeHg intrauterino e os impactos sobre desenvolvimento infantil até os dois anos de vida da criança indígena, o objetivo deste ensaio foi elaborar e apresentar um modelo teórico conceitual a partir de reflexões teórico-metodológicas que ilustram aspectos determinantes do neurodesenvolvimento nos primeiros mil dias de vida de crianças indígenas.

## Método

Esse estudo apresenta um ensaio, no qual o processo de elaboração e descrição de modelos teóricos foi desenvolvido com base nas sete etapas propostas por Souza Filho et al. ^22^. As etapas incluem: identificação e delimitação do objeto de estudo (etapa 1); resgate cognitivo e tempestade de ideias (etapa 2); representação do modelo teórico (etapa 3); revisão da literatura sobre o tema (etapa 4); estruturação do modelo teórico (etapa 5); submissão do modelo teórico a especialistas (etapa 6); e reestruturação e finalização do modelo teórico (etapa 7).

Para representação gráfica dos modelos teóricos aqui apresentados, foram utilizados como organizadores gráficos mapas conceituais, desenvolvidos por meio do software Drow.io (http://ww7.drow.io/). 

Os mapas conceituais foram adotados como método de representação gráfica por serem ferramentas que auxiliam a resumir o conhecimento e facilitam a aprendizagem significativa ^23^.

Durante o desenvolvimento do estudo, foi realizada uma revisão de literatura do tipo narrativa, para identificar e discutir modelos teóricos, conceitos, construtos, variáveis ou fatores relacionados ao atraso do neurodesenvolvimento em crianças menores de dois anos de idade, expostas ao metilmercúrio em comunidades indígenas.

A revisão de literatura foi realizada de março a dezembro de 2023, nas bases MEDLINE (via PubMed), SciELO e Google Scholar. Após a leitura do material bibliográfico identificado, as variáveis selecionadas foram categorizadas e analisadas para construção e descrição das dimensões que fizeram parte dos modelos teóricos. Foram utilizados blocos de diferentes cores para distinguir as categorias das variáveis nos modelos gráficos. 

Foi utilizado o instrumento *Checklist para Relato Teórico em Estudos Epidemiológicos* (CRT-EE) ^22^, composto por 15 itens que inclui uma coluna para marcação das respostas, de forma dicotômica (sim, não), acerca da inclusão de cada item no artigo.

## Exploração das variáveis no estudo

### Mercúrio e neurodesenvolvimento em crianças

Ao longo do neurodesenvolvimento humano, ocorre a proliferação e a migração de células neurais e o estabelecimento de conexões sinápticas para a formação de redes neurais complexas. Esse processo se inicia durante a gestação e nos primeiros anos de vida, e se consolida ao longo da infância e adolescência ^3^ ^,^ ^24^. O desenvolvimento de uma criança é um processo contínuo e dinâmico, que envolve a aquisição progressiva de novas habilidades e competências, que dependem da maturação do sistema nervoso ^3^. As causas das alterações do neurodesenvolvimento são classificadas de acordo com o período de exposição: pré-natal, perinatal ou pós-natal. A exposição pré-natal a substâncias tóxicas é um dos fatores envolvidos no atraso do neurodesenvolvimento, podendo afetar o desenvolvimento global e causar sequelas irreversíveis ^25^. A neurotoxicidade do MeHg ficou conhecida pelos estudos da exposição crônica em gestantes na Baía de Minamata, no Japão, em decorrência do consumo de peixes contaminados. Crianças nascidas de gestantes assintomáticas, expostas a níveis altos de MeHg, evoluíram com alterações do neurodesenvolvimento, caracterizadas pelo atraso motor e na fala, comprometimento das funções sensoriais, deficiência intelectual, convulsões, coma e morte ^25^.

Três estudos de coorte prospectivos, de larga escala, conduzidos de forma independente na Nova Zelândia, nas Ilhas Faroé (Dinamarca) e em Seychelles avaliaram os efeitos da exposição materna ao MeHg ^25^. Nos dois primeiros estudos, a exposição pré-natal ao MeHg mostrou-se associada à perda de pontos no quociente de inteligência (QI) e diminuição do desempenho em testes cognitivos, incluindo memória, atenção, linguagem e orientação espacial nas crianças. Por outro lado, no estudo conduzido em Seychelles, não foram observados efeitos adversos no desenvolvimento neuropsicológico e no QI. Apesar dos achados contraditórios, os estudos citados fornecem informações valiosas sobre os potenciais efeitos nocivos da exposição ao MeHg (mesmo em baixas doses) no desenvolvimento neurológico infantil. Enquanto os estudos da Nova Zelândia e das Ilhas Faroé sugerem um impacto negativo, o estudo em Seychelles não observou resultados adversos semelhantes ^25^. Os resultados heterogêneos podem ser devidos aos diferentes delineamentos dos estudos e ao padrão de consumo de peixes, por exemplo ^26^. 

Uma vez que já se sabe que polimorfismos em genes que metabolizam metais pesados no corpo humano influenciam na maneira como o organismo absorve, metaboliza, distribui e elimina o MeHg ^7^, diferenças genéticas entre as populações estudadas também podem explicar a heterogeneidade dos resultados.

Em um estudo que avaliou polimorfismos no gene GSTP1, envolvido na metabolização do MeHg no corpo humano, em 107 adultos do povo indígena Munduruku (Brasil), foi possível determinar uma associação estatística significativa entre variações nesse gene e a presença de sintomas neurológicos ^27^. Em conclusão, os autores recomendam o monitoramento genético da população como estratégia para identificar indivíduos com maior risco de desenvolver sinais e sintomas neurológicos. 

Em outro estudo ^(^ ^8^ , que analisou a associação entre a exposição ao MeHg e o neurodesenvolvimento infantil em 55 crianças indígenas Munduruku com menos de seis anos, por intermédio da aplicação do teste de Denver II, nove crianças apresentaram sinais de alerta. Os principais problemas identificados incluíram limitações na linguagem, comprometimento das habilidades motoras finas e grossas, bem como dos componentes pessoais e sociais do teste ^8^. O biomarcador utilizado para a exposição foi a dosagem de mercúrio total (HgT) em amostras de cabelo, como estimativa do MeHg ^28^. O nível médio de HgT nas amostras de cabelo das nove crianças com alterações no neurodesenvolvimento foi 7,34µg/g ^8^. Vale lembrar que a Agência de Proteção Ambiental dos Estados Unidos (EPA, acrônimo em inglês) ^29^ recomenda como dose de referência níveis inferiores a 1µg de HgT para cada grama de cabelo em pessoas com baixo consumo de peixe, incluindo gestantes. Shoerman et al. ^(^ ^30^ ^)^ advogam que a dose limite de segurança para fetos é entre 0,3 e 0,58µg/g no cabelo materno. Ou seja, as nove crianças Munduruku que apresentaram algum comprometimento no neurodesenvolvimento tinham níveis de mercúrio nas amostras de cabelo pelo menos sete vezes superiores à dose de referência estabelecida pela EPA. 

A despeito dos achados ilustrativos dos estudos revisados, a complexa interação entre os níveis de exposição ao MeHg, a susceptibilidade individual e os fatores ambientais no processo do desenvolvimento neurológico infantil requerem pesquisas mais aprofundadas.

### Variáveis envolvidas na gestação que alteram o neurodesenvolvimento do bebê

#### Exposição a substâncias tóxicas na gestação

Além da exposição ao MeHg, o uso de substâncias tóxicas como álcool e tabaco durante a gestação pode ter efeitos prejudiciais tanto para a mãe quanto para o feto. O consumo de álcool durante a gravidez aumenta o risco de aborto, parto prematuro, restrições no crescimento fetal e síndrome alcoólica fetal ^31^. Após absorvido pelo organismo materno, o álcool ultrapassa a membrana placentária e expõe o feto na mesma proporção que a gestante ^31^. O feto não tem todas as enzimas necessárias para metabolizar o álcool, fato que agrava a exposição fetal a esta substância. O álcool interfere no transporte de aminoácidos e ácidos graxos, promove vasoconstrição e risco de hipóxia fetal, além de aumentar o estresse oxidativo ^31^. Tais efeitos prejudicam o desenvolvimento fetal em várias áreas, sobretudo na organogênese e no crescimento fetal ^31^.

O tabagismo durante a gravidez resulta em diversos efeitos adversos, incluindo anomalias da placenta, retardo no crescimento pré-natal, aborto espontâneo, distúrbios cognitivos e doenças respiratórias ^32^. A exposição à nicotina tem sido associada a alterações cognitivas e de desenvolvimento psicomotor em crianças ^32^.

Na literatura especializada há diversos estudos que demonstram a associação entre a exposição ao MeHg e variáveis relacionadas ao nascimento. Um estudo na Tanzânia encontrou risco aumentado de natimorto (2,5 vezes) e presença de anomalias congênitas (2,2 vezes) em crianças com exposição pré-natal ao MeHg ^33^. Outros estudos sugerem que a exposição pode aumentar o risco de prematuridade, baixo peso ao nascer e diminuição do perímetro cefálico ^20^ ^,^ ^21^. 

Neste ensaio, foram consideradas na análise variáveis que potencialmente causam atraso no neurodesenvolvimento, independentemente da exposição pré-natal ao MeHg, como a atenção ao pré-natal, local e tipo de parto, história de violência, estresse e depressão materna, além de fatores socioeconômicos como idade e escolaridade materna.

#### Pré-natal

A partir do diagnóstico da gestação, a atenção pré-natal é recomendada ^34^. O pré-natal engloba ações que visam melhorar os indicadores maternos e infantis considerando os seguintes itens: desejo de engravidar, acesso a cuidados de saúde, uso de ácido fólico, cessação de tabaco e substâncias tóxicas, prevenção da depressão materna, promoção do ganho de peso materno segundo o estado nutricional pré-gestacional, ausência de infecções sexualmente transmissíveis, controle glicêmico em mulheres com diabetes pré-gestacional e não uso de medicamentos teratogênicos ^34^. Atenção especial deverá ser dispensada à gestante com maiores riscos, como aquelas com diagnóstico de doenças crônicas, histórico de complicações em gestações anteriores ou que vivem em condições de vulnerabilidade social ^35^.

#### Local e tipo de parto

O local do parto envolve vários aspectos, como segurança, intervenções médicas, conforto e preferências pessoais. Embora o parto domiciliar possa oferecer mais conforto para a parturiente, o parto hospitalar dispõe de equipamentos e intervenções essenciais em caso de emergências obstétricas, tornando-o mais seguro para gestantes de alto risco ^36^. Comparado à cesariana, o parto normal beneficia a função respiratória do recém-nascido pela compressão da caixa torácica e pela liberação de catecolaminas, além de facilitar o método pele a pele e a amamentação ^36^. Para a mãe, o parto normal proporciona melhor locomoção e alimentação, uma recuperação mais rápida e um processo ativo de maternagem ^36^.

#### Violência, estresse e depressão materna

Estudos indicam que as primeiras experiências biológicas e psicossociais na vida intrauterina, como a exposição à violência, ao estresse e à depressão materna, contribuem para o atraso do neurodesenvolvimento ^37^ ^,^ ^38^. A depressão durante a gestação pode afetar o desenvolvimento do eixo hipotálamo-hipófise-adrenal, expondo-o a níveis mais elevados do hormônio liberador de corticotrofina ^38^. A exposição a transtornos maternos de ansiedade ou depressão pode afetar o córtex pré-frontal fetal, responsável pelo controle das funções cognitivas. Acredita-se que esses efeitos se devam à exposição do feto a altos níveis de glicocorticoides ^38^. A presença de estresse na gestação aumenta ainda o risco de parto prematuro e bebê pequeno para a idade gestacional ^38^.

#### Alimentação da gestante e da criança

Durante o período da vida intrauterina e ao longo dos primeiros mil dias de vida o crescimento e o desenvolvimento são determinados por fatores genéticos, ambientais e sociais. Esse período é considerado o mais importante para a formação do ser humano ^5^. Nesse sentido, a assistência pré-natal e perinatal da gestante e do recém-nascido contribuíram para redução da mortalidade materna e infantil no século XX ^39^ ^,^ ^40^.

Mais recentemente, a alimentação da gestante, o aleitamento materno e a alimentação infantil (desde sua introdução, quanto a qualidade e a quantidade) passaram a ser considerados elementos definidores para o crescimento e o neurodesenvolvimento saudáveis ^5^.

Pescados consumidos durante a gestação e o nível de contaminação por MeHg em cada espécie de peixe ingerida pelas mães influenciam o processo de exposição e seus efeitos no sistema nervoso central dos fetos em desenvolvimento intrauterino ^13^. Em um estudo ^(^ ^10^ ^)^ que analisou 88 amostras de pescados provenientes da Terra Indígena Munduruku, na bacia do Rio Tapajós, no Pará, foi possível constatar que as maiores concentrações de mercúrio foram observadas em peixes que consomem outros peixes, em comparação com os peixes predominantemente herbívoros. 

A carência de aporte nutritivo para cada faixa etária, como o déficit protéico calórico, ou o déficit de nutrientes como o ferro, trazem prejuízos à curto, médio e longo prazos ^39^. A anemia afeta o metabolismo energético, o crescimento, a função imunológica, o desenvolvimento cognitivo e a função cardiovascular ^39^.

Por sua vez, o aleitamento materno tem inúmeros benefícios conhecidos na literatura ^40^. Entretanto, em contextos de exposição ao mercúrio no período pré e perinatal, o MeHg pode ser excretado pelo leite materno, sendo transferido à criança após o nascimento ^41^. Por outro lado, o aleitamento materno é um fator protetor para o neurodesenvolvimento e fonte de ácidos graxos poli-insaturados das séries ômega-3. Ressaltamos que não foram encontradas referências que desaconselham o aleitamento materno em nutrizes com exposição ao MeHg.

Outro elemento importante na alimentação é o selênio. Esse micronutriente é componente-chave de várias selenoenzimas antioxidantes, que ajudam a proteger as células contra danos oxidativos ^42^. A castanha-do-pará constitui importante fonte de selênio para as populações amazônicas. Em dois estudos transversais realizados na Região Amazônica, as populações indígenas apresentaram níveis mais elevados de selênio em comparação com as populações controle ^43^ ^,^ ^44^. Populações que vivem em áreas com baixos níveis de selênio podem ser mais suscetíveis aos efeitos tóxicos do mercúrio, especialmente quando há uma exposição significativa ao metal ^44^.

**Figura 1:**
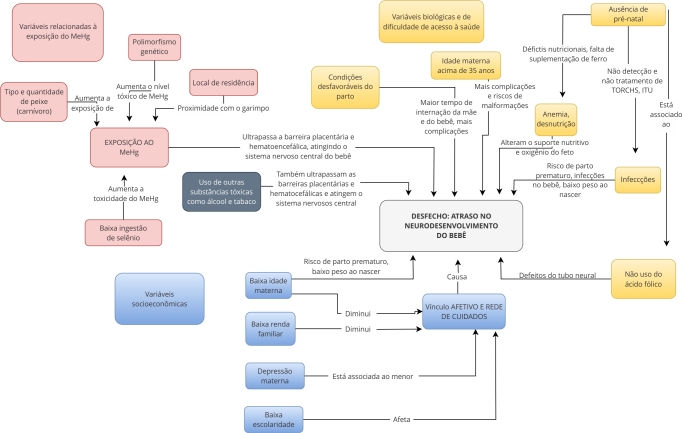
Modelo teórico conceitual com as relações observadas entre as variáveis que podem estar associadas ao atraso do neurodesenvolvimento da criança durante a gestação e o parto.

Os pontos acima destacados demonstram que uma abordagem ampla e integrada, envolvendo tanto os fatores biológicos quanto socioeconômicos e ambientais, tem impacto significativo na saúde e no desenvolvimento infantil ^37^. Na Figura 1 visualizam-se graficamente as variáveis que podem estar associadas ao atraso de neurodesenvolvimento da criança relacionadas à gestação e ao parto.

MeHg: metilmercúrio. Fonte: elaborado pelos autores.

O modelo teórico conceitual da Figura 1 apresenta em vermelho as variáveis relacionadas à exposição materna ao MeHg. O consumo de peixe (peixes maiores, carnívoros e ingeridos com maior frequência), o local da residência (mais próximo ao garimpo e maior influência da violência, uso de substâncias tóxicas, infecções sexualmente transmissíveis), o polimorfismo genético (genes envolvidos no metabolismo do mercúrio), a baixa ingestão de selênio (presente na castanha-do-pará), aumentam a exposição e a sua toxicidade. O local de residência próximo ao garimpo, além de aumentar a exposição ao MeHg, pode sofrer mais influências como violência e conflitos. O MeHg atravessa a barreira placentária e hematoencefálica atingindo o sistema nervoso do feto ainda no útero materno, provocando alterações do neurodesenvolvimento. O uso de outras substâncias tóxicas, como o álcool e o tabaco, exerce papel semelhante (cor cinza). Além disso, as variáveis descritas em azul: baixa idade materna (menor de 15 anos), baixa renda familiar, depressão materna e baixa escolaridade materna diminuem o vínculo afetivo, a rede de cuidados e o ambiente favorável ao neurodesenvolvimento da criança. 

A baixa idade materna está associada ao baixo peso ao nascer e à prematuridade, que impactam o neurodesenvolvimento ^45^ ^,^ ^46^. As variáveis biológicas na cor amarela indicam que, durante o período pré-natal, são conduzidos testes para identificar doenças infecciosas, como toxoplasmose e sífilis, que podem ser tratadas com intervenções farmacológicas. O diagnóstico e tratamento das infecções do trato urinário diminuem a ocorrência do parto prematuro e o baixo peso ao nascer ^35^. Além disso, o uso do ácido fólico no período pré-concepcional previne a malformação do tubo neural no feto ^35^. Durante o pré-natal, a suplementação de ferro é orientada para prevenir a anemia materna, que pode causar dano neural ^35^. A idade materna acima de 35 anos aumenta o risco de doenças cromossômicas e complicações na gravidez ^35^.

Adicionalmente, condições de parto desfavoráveis podem causar mais hemorragias, tocotraumatismos, hipóxia no bebê e infecções, aumentando a internação da mãe e do recém-nascido ^36^.

#### Hipóxia

A asfixia perinatal envolve a interrupção do fluxo sanguíneo placentário, o que leva à hipóxia fetal, hipercapnia e acidose ^47^. A falta de oxigênio leva à produção de espécies reativas de oxigênio, que causam danos oxidativos e inflamação nas células cerebrais, que podem levar a sequelas neurológicas permanentes ^47^. 

#### Prematuridade

No Brasil, cerca de 11% dos partos são prematuros, sendo a maior causa de mortalidade infantil no país ^48^. A prematuridade está associada a déficits neurológicos ^45^. Bebês prematuros apresentam maior probabilidade de desenvolver lesões cerebrais, como hemorragia intraventricular e leucomalácia periventricular devido à imaturidade fisiológica e respostas limitadas à exposição precoce ao ambiente extrauterino. Bebês prematuros podem experimentar atrasos na fala, cognição e habilidades sociais, sendo a menor idade gestacional fortemente associada ao atraso do neurodesenvolvimento ^49^.

#### Baixo peso ao nascer e retardo do crescimento intrauterino

O baixo peso ao nascer é caracterizado por peso de nascimento menor a 2.500g. Estima-se que 15% a 20% dos recém-nascidos em todo o mundo tenham baixo peso ao nascer. No Brasil, a proporção varia entre as regiões, com taxas entre 7,2% e 8,4% ^46^. O baixo peso ao nascer está associado a riscos significativos para a saúde como morbidade e mortalidade durante o primeiro ano de vida, além de maior incidência de distúrbios neurocognitivos. Os padrões de restrição de crescimento durante a vida fetal podem afetar outros sistemas no organismo, como cardiovascular e endócrino, com aumento do risco de desenvolvimento de hipertensão arterial, infarto do miocárdio e diabetes mellitus tipo 2 na vida adulta ^41^ ^,^ ^50^. 

#### Outras causas envolvidas

O atraso do neurodesenvolvimento pode acontecer por outras causas, incluindo genéticas (mutações em um único gene, doenças cromossômicas ou metabólicas) ^51^; e multiparidade, uma vez que bebês nascidos de partos múltiplos têm maior probabilidade de desenvolver dano cerebral do que bebês nascidos de partos únicos. Esse risco aumentado foi atribuído principalmente à maior prevalência de prematuridade em partos múltiplos ^51^.

Ainda podem ser citadas outras causas como traumas e acidente vascular cerebral, e as infecções ocorridas durante a gravidez (corioamnionite, sífilis, rubéola, citomegalovirus, toxoplasmose), no parto (sepsis neonatal) ou na infância (encefalites e meningites) ^51^. 

#### Calendário vacinal

A vacinação de crianças desempenha um papel fundamental na promoção da saúde, pois previne doenças individual e coletivamente, colaborando para o controle/erradicação ^52^. Desde 2016, porém, é observada queda da cobertura vacinal no Brasil, apesar do Programa Nacional de Imunizações ser reconhecido como um dos mais completos do mundo ^52^. 

#### Doenças detectadas no teste de triagem neonatal

A triagem neonatal é realizada em recém-nascidos para identificar precocemente doenças genéticas e metabólicas que podem causar problemas de saúde graves se não forem tratadas precocemente. O teste do pezinho é obrigatório no Brasil, sendo uma parte fundamental dos cuidados de saúde neonatal ^53^. 

#### Acesso à saúde e educação

Crianças que não têm acesso adequado a cuidados de saúde, nutrição, educação de qualidade e apoio emocional, estão em maior risco para atraso do neurodesenvolvimento ^54^. Países que implementaram programas abrangentes de desenvolvimento infantil, que incluem cuidados em saúde, nutrição, estimulação e educação, alcançaram resultados significativos e duradouros ^55^. Essas evidências estão alinhadas com a compreensão científica de que os primeiros anos de vida desempenham um papel crucial no desenvolvimento de habilidades essenciais para a formação da população economicamente produtiva. 

#### Variáveis relacionados à saúde da criança indígena

A saúde de populações indígenas no Brasil é marcada por relevante desigualdade nos principais indicadores, com destaque para as desproporcionais taxas de mortalidade infantil ^7^. A precária articulação entre o modelo biomédico vigente e a medicina indígena impõe desafios adicionais à assistência à saúde ^15^. Estudo conduzido com o povo Guarani revelou altas prevalências de baixo peso ao nascimento e prematuridade. Esses desfechos foram associados à idade materna, ao estado nutricional, à paridade, às condições de moradia e a poluição domiciliar. Segundo os autores ^56^, o acesso e a qualidade do pré-natal podem melhorar os resultados encontrados, impactando positivamente a saúde da população.

Em um estudo nacional com 3.967 mulheres indígenas de 14 a 49 anos, 86,6% receberam assistência pré-natal, porém apenas 30% iniciaram no primeiro trimestre e 16% realizaram sete ou mais consultas. Exames pré-natais foram solicitados para 53% e sulfato ferroso prescrito para 44%. Esses índices revelam desigualdades étnico raciais ^7^ ^,^ ^57^. Outro estudo encontrou inadequações da assistência ao pré-natal em indígenas, com desigualdades de acordo com a etnia e local de moradia ^58^.

Populações indígenas frequentemente residem em áreas rurais e de florestas, reconhecidamente endêmicas para malária. A maior exposição aos mosquitos transmissores resulta em um maior risco de desenvolver a doença, sobretudo em mulheres e crianças. O acesso restrito a cuidados médicos adequados, ao diagnóstico precoce e ao tratamento oportuno, associado às precárias condições de vida e a falta de medidas de prevenção contribuem para a disseminação da malária nessas comunidades ^59^.

Em estudo ^(^ ^60^ ^)^ que analisou hospitalizações em crianças indígenas Guarani, foi possível observar tempo de internação elevado e alta frequência de internações por enfermidades que poderiam ser prevenidas pela atenção primária. Considerando as infecções respiratórias, os autores relatam falha no tratamento domiciliar, dificuldade de acesso ao serviço e a medicamento na localidade, além de barreiras linguísticas ^60^. 

**Figura 2:**
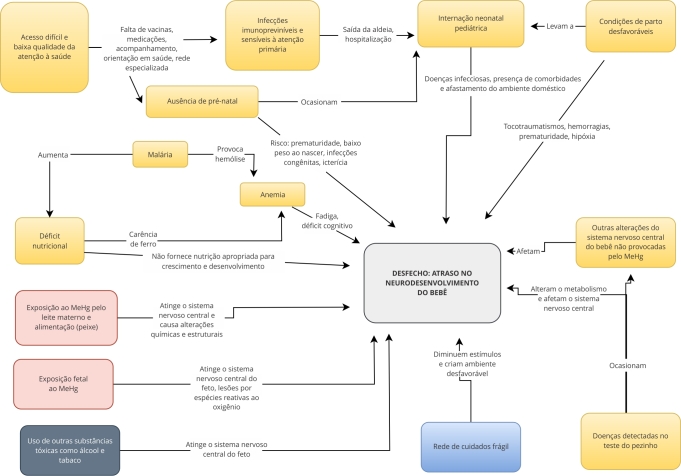
Modelo teórico conceitual com as relações observadas entre as variáveis relacionadas ao atraso do neurodesenvolvimento da criança indígena.

O modelo teórico conceitual da Figura 2 representa a rede causal de exposição da criança indígena ao MeHg (vermelho) relacionada à ingestão de leite materno ou peixe contaminados, podendo resultar no atraso do neurodesenvolvimento. Além disso, a exposição ao álcool e tabaco que são excretados pelo leite materno, igualmente afetam o feto durante a gestação. As demais variáveis, em amarelo, estão relacionadas ao retardo no neurodesenvolvimento devido às limitações de acesso ou à baixa qualidade da atenção à saúde, bem como às infecções imunopreveníveis e alguns agravos sensíveis à atenção primária. Similarmente, as doenças detectadas pelo teste do pezinho e as alterações congênitas ou adquiridas do sistema nervoso da criança não relacionadas à exposição ao MeHg afetam o neurodesenvolvimento, assim como a desnutrição e a anemia. Ademais, a malária causa anemia por hemólise, contribuindo para agravamento do estado nutricional.

MeHg: metilmercúrio. Fonte: elaborado pelos autores.

Em síntese, compreender as relações entre variáveis associadas ao neurodesenvolvimento infantil e a exposição ao MeHg, considerando a presença de possíveis efeitos confundidores e demais questões relacionadas à inferência causal, converte-se numa questão para a correta tomada de decisão nos modelos teóricos existentes, bem como em seu processo de interpretação.

## Reflexão

A elaboração de modelos teóricos sobre os impactos da exposição ao MeHg no neurodesenvolvimento de crianças indígenas mostrou-se como ferramenta efetiva para ampliar o conhecimento nessa área temática. A inclusão de um vasto conjunto de variáveis nas análises e nas reflexões aqui apresentadas possibilitou a identificação de fatores que podem influenciar o neurodesenvolvimento das crianças, bem como permitiu explorar relações complexas como, por exemplo, a interação entre exposição ao MeHg e o peso ao nascer e sua influência sobre o desfecho em investigação. Além de variáveis clínicas, os modelos explicitados neste ensaio consideraram ainda aspectos socioeconômicos como escolaridade e renda das famílias, bem como o acesso aos serviços de atenção pré-natal e ao parto. 

Entretanto, algumas limitações foram identificadas no processo de elaboração do modelo. Inicialmente, ressalta-se a escassez de dados na literatura sobre neurodesenvolvimento em populações indígenas, seja no Brasil ou no exterior. Uma vez que os dados do Subsistema de Saúde Indígena não são abertos para consulta pública, obter informações das condições de nascimento, de hospitalizações da criança e da mãe, e da assistência à saúde nas aldeias constituem um desafio. Para contornar essa limitação, foram debatidos achados do *Inquérito Nacional de Saúde e Nutrição Indígena* bem como diferentes estudos conduzidos com populações indígenas da Amazônia. 

A escassez de estudos sobre os efeitos do metilmercúrio no neurodesenvolvimento de povos indígenas é um desafio, mas também uma oportunidade para este ensaio, que busca contribuir para a ampliação do entendimento sobre o tema.

Esta pesquisa focou variáveis relevantes para a realidade indígena, contando com bases biológicas plausíveis e mensuráveis em estudos de campo. Contudo, a elaboração de modelos teóricos apresenta limitações inerentes, que incluem a possível omissão ou exclusão de variáveis no próprio modelo, ou nas posteriores análises estatísticas. A ausência de variáveis pertinentes ao contexto estudado pode levar a uma análise superficial, dificultando a replicação e a validação dos resultados por outros pesquisadores.

Por se tratar de uma atividade ilegal, não há estatísticas oficiais no Brasil sobre o adoecimento e mortes decorrentes da contaminação por mercúrio em povos indígenas, tampouco estimativas das quantidades de mercúrio utilizadas na garimpagem em terras indígenas.

Em pesquisas envolvendo populações etnicamente diferenciadas, idealmente os modelos teóricos deveriam considerar fatores culturais que afetam positivamente o neurodesenvolvimento, tais como habilidades específicas observadas em contextos indígenas, bem como cuidados comunitários com as crianças, temas não explorados neste ensaio. Esse cuidado visa evitar generalizações inadequadas, uma vez que crianças indígenas frequentemente participam de atividades realizadas em grupos, ao ar livre, e em estreita conexão com a natureza ^61^. O uso de telas e dispositivos eletrônicos é limitado, e as crianças se envolvem em atividades lúdicas que simulam responsabilidades sociais de adultos, como manejo de arcos e flechas, facões, preparo de alimentos e cuidado com irmãos menores. Essas práticas são rotineiras nas aldeias indígenas e desempenham um papel significativo no desenvolvimento infantil ^61^.

Apesar das limitações descritas acima, destacam-se alguns pontos fortes. Os modelos teóricos aqui apresentados, além de inéditos na literatura, oferecem uma perspectiva ampliada sobre a questão, apontando reflexões para o aperfeiçoamento da assistência materno-infantil, no contexto indígena. Além de contribuir para o embasamento teórico, trazem fundamentos para o aprimoramento da atenção ofertada pelas equipes de saúde. Nos programas de atenção pré-natal e de crescimento e desenvolvimento infantil podem ser implementadas ações que incluem a dosagem dos níveis de mercúrio no cabelo da mulher e do bebê, bem como orientações sobre o consumo seguro de peixes pela gestante, com preferência para peixes menores e não carnívoros, além do aumento do consumo de castanhas. 

Adicionalmente, as figuras apresentadas neste ensaio podem ser utilizadas didaticamente em sessões de educação em saúde realizadas por profissionais junto às comunidades indígenas. 

Uma das principais vantagens dos modelos teóricos é esclarecer as hipóteses subjacentes sobre a exposição e o desfecho estudado, permitindo uma análise crítica de suas implicações e conclusões. Isso ajuda a identificar possíveis fontes de viés e incerteza, facilitando a compreensão dos fatores que influenciam o atraso no neurodesenvolvimento infantil ^62^.

## Conclusão

O modelo teórico aqui elaborado incluiu fatores clínicos, biológicos, sociais, econômicos, culturais e ambientais que afetam o neurodesenvolvimento infantil, demonstrando coerência com resultados de estudos que apontam que as populações indígenas contemporâneas enfrentam múltiplos desafios para ter garantido seu acesso a serviços públicos essenciais e a direitos humanos universais. A exposição a condições adversas como falta de saneamento, acesso limitado à saúde e educação, baixa renda, anemia, desnutrição e exposição a contaminantes químicos prejudicam o desenvolvimento infantil, afetando habilidades cognitivas, motoras e sociais. 

Ademais, somam-se às condições adversas acima mencionadas a presença de garimpos ilegais em terras indígenas, e a contaminação dos recursos naturais e das fontes de alimento pelo mercúrio, agravando a situação. A exposição de gestantes e crianças ao MeHg resulta em danos neurológicos irreversíveis, comprometendo a qualidade de vida de gerações presentes e futuras, com reflexos negativos para toda a sociedade.

Apesar das limitações inerentes aos modelos teóricos, as potencialidades deste ensaio incluem a originalidade do modelo aqui apresentado, e sua contribuição para o aperfeiçoamento da assistência materno-infantil indígena. 

Em conclusão, para reduzir o atraso no neurodesenvolvimento sugere-se a implementação de programas de saúde pública adaptados à realidade local das comunidades indígenas investigadas, o fortalecimento das ações de educação em saúde nas comunidades, o acesso equitativo a serviços de saúde de qualidade, e a conscientização sobre os riscos ambientais associados à exposição a substâncias tóxicas e seus prejuízos. Por fim, diante das invasões de garimpos ilegais e suas consequências sociais, ambientais e para saúde da população não se pode perder de vista a importância da proteção dos territórios tradicionais e da garantia da soberania dos povos que lá vivem.
